# Attention and awareness each influence amygdala activity for dynamic bodily expressions—a short review

**DOI:** 10.3389/fnint.2012.00054

**Published:** 2012-08-02

**Authors:** Beatrice de Gelder, Ruud Hortensius, Marco Tamietto

**Affiliations:** ^1^Cognitive and Affective Neuroscience Laboratory, Tilburg UniversityTilburg, Netherlands; ^2^Brain and Emotion Laboratory Leuven, Division of Psychiatry, Department of Neurosciences, KU LeuvenLeuven, Belgium; ^3^Department of Cognitive Neuroscience, Faculty of Psychology and Neuroscience, Maastricht UniversityMaastricht, Netherlands

**Keywords:** amygdala, attention, awareness, bodily expressions, emotion, dynamic stimuli

## Abstract

The amygdala (AMG) has long been viewed as the gateway to sensory processing of emotions and is also known to play an important role at the interface between cognition and emotion. However, the debate continues on whether AMG activation is independent of attentional demands. Recently, researchers started exploring AMG functions using dynamic stimuli rather than the traditional pictures of facial expressions. Our present goal is to review some recent studies using dynamic stimuli to investigate AMG activation and discuss the impact of different viewing conditions, including oddball detection, explicit or implicit recognition, variable cognitive task load, and non-conscious perception. In the second part, we sketch a dynamic dual route perspective of affective perception and discuss the implications for AMG activity. We sketch a dynamic dual route perspective of affective perception. We argue that this allows for multiple AMG involvement in separate networks and at different times in the processing streams. Attention has a different impact on these separate but interacting networks. Route I is engaged in early emotion processing, is partly supported by AMG activity, and is possibly independent of attention, whereas activity related to late emotion processing is influenced by attention. Route II is a cortical-based network that underlies body recognition and action representation. The end result of route I and II is reflexive and voluntary behavior, respectively. We conclude that using dynamic emotion stimuli and a dynamic dual route model of affective perception can provide new insights into the varieties of AMG activation.

## Introduction

The role of the amygdala (AMG) in processing behaviorally salient stimuli is widely documented in many animal and human studies. A variety of affective functions have been attributed to AMG activity including immediate perception of affective stimuli, learning, and conditioning as well as emotional memory (Phelps and LeDoux, 2005). The AMG is also involved in modulating cognitive functions as well as behavior and has many connections to brain areas directly involved in behavioral output (Mosher et al., 2010). As early as 1888, rhesus monkeys with a temporal cortex lesion (including the AMG) showed significant social and emotional changes. The matter was later studied systematically by Bucy and Klüver (1955) and shortly after Weiskrantz (1956) showed that bilateral ablation of the AMG was sufficient to induce the symptoms associated with the Kluver and Bucy syndrome. For a while, research focused primarily on behavioral studies in rodents (LeDoux, 1996). Human investigations of the AMG functions have been guided by and followed in the steps of the animal research findings. It is worth pointing out that the early animal studies used behavioral criteria to assess the influence of AMG on emotion processing. This may be just one of the difficulties that, combined with limited knowledge of limbic system anatomy in humans, make generalizations complicated.

Since the beginning of functional magnetic resonance imaging (fMRI) studies in humans, the AMG has also been the centerpiece of many reports in neurotypical controls (e.g., Morris et al., 1998) and various clinical groups or even populations with personality disorders and patients with focal brain damage (Morris et al., 2001; Siebert et al., 2003). Several researchers have provided extensive insights into the neuroanatomy of the AMG and the neighboring structures (Aggleton and Mishkin, 1986; Swanson and Petrovich, 1998). However, besides a general agreement on the central role of the AMG, many aspects of its functional significance for human behavior still await further clarification.

A striking finding is that the AMG exerts some of its functions of paradigmatic cognitive processes such as attention or perception either when the observer is fully aware of the nature and content of the stimulus or, alternatively, in implicit settings. This happens, for example, in settings in which visual awareness is lacking (Whalen et al., 1998; Morris et al., 1999; Liddell et al., 2005) or when the content of the stimulus is irrelevant for the task at hand (Vuilleumier et al., 2001). In the present context where these distinctions are central, we contrast studies reporting AMG activity under conditions of full awareness and normal vision of the stimulus with studies showing AMG activity under conditions in which stimulus awareness is lacking either because of sensory or attentional manipulation or because of brain damage.

The overwhelming majority of human fMRI studies that report AMG activation have used pictures of facial expressions. As a consequence, the findings obtained with facial expressions have dominated our view of AMG functions in this past decade. But this relatively limited basis is likely to confine our understanding of the role of the human AMG. New perspectives on human emotional behavior and new technologies have now made possible to present much more realistic and rich pictures to participants in fMRI experiments (e.g., Grosbras et al., 2012). For example, stimulus duration in video clips and the presence of movement are two main features among the factors that may lead to a different picture of the relation between cognitive, task dependent, factors and affective information on AMG activation.

The theoretical vantage point from which this review proceeds has been formulated by a few authors over the last decades in both animal and human emotion research and has been discussed in different contexts, mostly related to face processing. This theoretical perspective is variedly referred to as a dual route model of affective stimulus perception (LeDoux, 1996; Vuilleumier, 2005; Tamietto and de Gelder, 2010; Garrido et al., 2012), more specifically we have referred to it as dynamic dual route model of face perception (de Gelder and Rouw, 2001; de Gelder et al., 2003). A similar approach has not yet been developed systematically as a framework for whole body expressions perception.

We believe it makes a difference for the understanding of the relation between attention, consciousness, and the AMG whether one adopts a linear or a parallel dual route model of face or body processing. In research from our own lab we have provided evidence for this dynamic dual route perspective while at the same time including in it the notion that both routes are operational in parallel (e.g., de Gelder et al., 2003). Based on our work with blindsight patients we have also stressed the notion that attention and task related factors are different from sensory unawareness and have different effects on consciousness (Tamietto and de Gelder, 2010). As we will point out below, the contrast between these routes must not be viewed as paralleling the familiar distinction between implicit versus explicit, with or without attention or non-conscious versus conscious. The importance of this model may appear more clearly when dynamic stimuli are used since dynamic images presumably trigger more or/and partly different processes in online emotion perception.

In this brief review we discuss the state of the art concerning the role of the AMG in experiments that instead of using short presentations of static stimuli have presented participants with full naturalistic videos. Using this kind of stimuli can provide us with a more detailed view of the human AMG functions. In the first part we review studies from our lab using dynamic bodily expressions to investigate AMG activation under oddball detection, with either explicit or implicit recognition demands, with variable cognitive task load, and, finally, under conditions of non-conscious perception (see Table 1 for an overview of the discussed studies and Supplemental Online Materials for examples of the stimuli used in these studies). In the second part we spell out how controversies concerning the role of AMG in affective perception can be put in perspective when one adopts a dynamic dual route perspective on affective perception that allows for AMG involvement in separate networks and at different times in the processing streams.

**Table 1:** **Overview of discussed studies using dynamic bodily expressions**.

**Study**	**Task**	**Stimuli**	**Amygdala localization**	Talairach coordinates^***^	**Results for the amygdala**
Grèzes et al., 2007	Oddball detection	Person opening a door in a fearful or neutral manner and scrambles^*^	Whole brain analysis	Right: 27/–3/–20	Bodies > scrambles
Grèzes et al., 2009	Oddball detection	Person opening a door in a fearful or neutral manner^*^	Whole brain analysis	Right: –35/0/–14	–No AMG activation for fearful > neutral in ASD group
					–Weaker connections between the AMG and STS, IFG, and PM in the ASD group.
Kret et al., 2011a	Oddball detection	Angry, fearful, or neutral facial and bodily expressions	Functional localizer^**^	Right: 17/–6/–10	Faces > bodies
				Left: –17/–8/20	
Kret et al., 2011b	Oddball detection	Angry, fearful, or neutral facial and bodily expressions	Functional localizer^**^	Right: 17/–6/–10	Male participants > female participants for faces > bodies contrast
				Left: –17/–8/20	
Kret et al., 2011c	Oddball detection	Angry, fearful, or neutral facial and bodily expressions	Functional localizer^**^	Right: 21/–10/–6	Negative correlation between negative affectivity and threatening faces and bodies > neutral faces and bodies contrast
Pichon et al., 2008	Oddball detection	Person opening a door in an angry or neutral manner and scrambles^*^	Whole brain analysis and sphere	Body > scrambles	–Bodies > scrambles
				Right: 19/–4/–8	–Anger > neutral
				Left: –33/–1/–17	
				Anger > neutral	
				Right: 27/–3/–18	
Pichon et al., 2009	Emotion-naming	Person opening a door in an angry, fearful, or neutral manner	Whole brain analysis	Left: –18/–8/10	–Threatening > neutral
					–Positive correlation between fear recognition and fear > neutral contrast
Pichon et al., 2012	Emotion-naming and color-naming	Person opening a door in an angry, fearful, or neutral manner	Whole brain analysis	Right: 29/–7/–17	–Threatening > neutral in emotion-naming
				Left: –33/–5/–15	–Deactivation in the color-naming task
Pouga et al., 2010	Oddball detection	Person opening a door in a fearful or neutral manner^*^	Whole brain analysis	Right: 17/–8/–17	–Fear > neutral
				Left: –28/–3/–19	–Negative correlation between difficulty identifying emotions and fear > neutral contrast
Sinke et al., 2010	Emotion-naming and color-naming	Teasing or threatening social interaction and scrambles	Anatomically defined for individual subjects	Right AMG: 18 ± 2.4/–5 ± 3.6/–16 ± 1.7	–Deactivation in both the emotion-naming and color-naming task
					–Less deactivation for threatening social interactions regardless of task condition
Sinke et al., 2012	Easy or hard color-naming with focus on aggressor or passive victim	Threatening social interaction between an aggressor and passive victim	Group mask	Left AMG: –19/–7/–13	–Deactivation in both the easy and hard color-naming task
					–Less deactivation when focus on aggressor, especially in easy color-naming task
Van den Stock et al., 2011	Oddball detection	Person opening a door in an angry or neutral manner	Anatomically defined	Right: 19/–2/–5	–Anger > neutral only for non-conscious perception
				Left: 22/–7/–6

* Static stimuli were also shown. No difference between static and dynamic stimuli in terms of AMG activation.

** AMG was localized using a separate localizer run with face, body, tool, and house stimuli using a face > house contrast.

*** MNI coordinates were transformed to Talairach coordinates by using the Nonlinear Yale MNI to Talairach Conversion Algorithm (Lacadie et al., 2008).

The review will highlight the complex way in which emotional stimuli are processed in the brain and the interplay between emotion and cognition. Specifically, we will focus on the interaction between emotion and attention, as the latter can be considered a typical cognitive function. In fact, a central role of attention is to modulate sensory processing, for example, by increasing the firing rate in primary sensory areas or by enhancing behavioral performance. In recent years, such functions have also been reported during the processing of emotional stimuli and have been related to the activity of the AMG. Thus, converging evidence is pointing to the AMG as a central hub in the dynamic interplay between emotion and cognition and makes the study of the functional and anatomical properties of this structure a paradigmatic case for the study of emotion–cognition interaction.

## Different task conditions and amygdala activation

### Amygdala activation under oddball detection

Experiments using oddball detection provide valuable insight when affective processing for new classes of stimuli are investigated and they have the extra advantage of allowing a closer comparison with animal data that rarely use complex evaluative tasks. Our first study with video clips used a passive viewing paradigm requiring participants simply to detect the oddball stimuli presented upside-down (Grèzes et al., 2007). Stimuli consisted of 3 s long video clips showing a person facing the camera and opening a sliding door in an emotional or neutral manner. An important aspect of this study is that it comprised video clips as well as still images taken from the same video clips and shown for the same duration. The main finding for our present question was that viewing the action, whether static or dynamic and independently of whether the expression was fearful or neutral enhanced right AMG activity relative to scrambles. The fact that the right AMG is more activated in all conditions where a whole bodily action is contrasted with its scrambled counterpart may in part be related to the type of action used here, which always shows an other directedness. It may also be the case that even the neutral door opening action is spontaneously interpreted as having an affective significance.

Pichon et al. (2008) repeated this same design but using anger instead of fear expressions. Again, relative right AMG activation was increased regardless of explicit movement (dynamic vs. static) and emotion (anger vs. neutral). Other regions were also activated similar to observations obtained in the previous study with dynamic fearful bodily expressions (Grèzes et al., 2007), as well as a previous study using static fearful bodily expressions (de Gelder et al., 2004). In the perception of dynamic body expressions of anger, brain regions that are coupled with autonomic reactions and motor responses related to defensive behaviors, such as the ventromedial prefrontal cortex, the temporal pole, and the premotor cortex (PM) were also activated.

Another paper by Kret et al. (2011a) directly investigated the similarities and differences between the processing of dynamic threatening facial and bodily expressions. Results showed that in this comparison right AMG activation was highest for dynamic facial expressions compared with bodily expressions. In line with the two previous studies discussed above, no difference was observed between threatening or neutral expressions. Additional analysis showed that male participants drove the difference in AMG activation between dynamic facial and bodily expressions (Kret et al., 2011b). While not statistically significant, AMG activation was highest when male participants observed female faces.

Personality factors or psychiatric disorders may also influence AMG activity. Kret et al. (2011c) looked at the role of negative affectivity in the processing of dynamic threatening facial and bodily expressions. A negative correlation between left AMG activity for threatening versus neutral faces and bodies and negative affectivity was observed (Kret et al., 2011c). In other words, people high on negative affectivity (the experience of negative emotions across time and situations) have less relative AMG activation when processing threatening facial and bodily expressions. Using the same task and dynamic stimuli as the first study from our lab (Grèzes et al., 2007), we recently showed that relative AMG activation levels do differentiate between high and low alexithymia, a personality trait associated with deficits in emotional reactivity and regulation (Pouga et al., 2010). A negative correlation was found between the level of difficulty to identify one's emotional experiences and relative right AMG activation in response to fearful stimuli. In line with this finding, adults with autism show no differential AMG activation in the perception of fearful actions (Grèzes et al., 2009). Interestingly, in the same study weaker connections between the AMG and superior temporal sulcus (STS), inferior frontal gyrus (IFG), and PM were found.

### Explicit vs. implicit emotion recognition

To further investigate AMG activation under different task conditions, Pichon et al. (2009, 2012) used the same stimuli as in previous studies (Grèzes et al., 2007; Pichon et al., 2008), namely angry, fearful, and neutral actions. Importantly, here a different task than simple oddball detection was used. The goal was to compare the pattern of brain activity in the condition of explicit recognition (naming of the represented emotion) with that observed in the alternative implicit condition where subjects had to attend to and name a colored dot. The results of this study were reported in two papers. In the first one the comparison between the neurofunctional signature of fear and that of anger under explicit task conditions was described (Pichon et al., 2009). The interesting result is that both emotion categories trigger stronger AMG activity compared with the neutral condition. We conjectured that this reflects the fact that anger as well as fear cues function as threat signals. On the other hand, we did observe an important difference at the level of AMG activity between fear and anger conditions when considering the role of the AMG in recognizing dynamic emotion actions. Recognition performance for fearful stimuli was significantly correlated with relative AMG activation for fearful expressions.

The results obtained in the comparison between emotion-naming (explicit) and color-naming (implicit) conditions allow us to enter the debate on the role of attention in AMG activation. In the literature there is a longstanding debate if implicit or pre-attentive processing of emotional stimuli triggers AMG activation. Two contradictory lines of research are described (for a review see Pessoa, 2005; Vuilleumier, 2005). Vuilleumier et al. (2001) showed that AMG activation in response to static fearful facial expressions is relatively independent of attentional demands (or less modulated by attention than other emotion-sensitive structures), whereas Pessoa et al. (2002) reported that attention to the affective stimulus is a prerequisite for AMG activation in response to static fearful and happy facial expressions. Both studies used dual-task paradigms in which they presented static emotional faces together with different unrelated stimuli and contrasted AMG activation to attended faces with that to unattended faces. Using an event-related fMRI design, the task of Vuilleumier et al. (2001) involved matching two faces similar in emotional expression (attended face) or two houses (unattended face) in a stimulus display, whereas participants in the Pessoa et al. (2002) study asked participants to judge in alternating blocks the gender of the face (attended face) or the orientation of bars (unattended face).

Using dynamic stimuli we can provide additional information in the debate on automaticity of the AMG response to threatening social information. In contrast to the observation of AMG activation to both angry and fearful social actions in the explicit recognition task (Pichon et al., 2009), no increase in AMG activation was found under implicit task demands for both angry and fearful stimuli (Pichon et al., 2012). Using a similar task, but incorporating a more social dimension, Sinke et al. (2010) used threatening or teasing social interactions between pairs of actors. Interestingly, under both task conditions deactivation in the right AMG was observed. However, deactivation was less pronounced for the threatening social interaction in both the explicit and implicit task.

These results show a complex pattern. The disengagement of the AMG (as suggested by deactivation) under implicit conditions (Sinke et al., 2010; Pichon et al., 2012) is consistent with the literature suggesting a mediating effect of attention on AMG activity to affective stimuli (e.g., Pessoa et al., 2002). To explain their effects, Pichon et al. (2012) distinguish between two subcortico-cortical networks. The first is a PM-hypothalamus-periaqueductal grey (PAG) network which functions independent of task demands and attention, while the second network, partly formed by the AMG and areas in the temporal cortex (STS, fusiform gyrus), is influenced by task demands. During a complex and challenging task multiple sources compete for attention and a successful strategy requires disregarding potentially distracting information. However, while affective information might be irrelevant to the task, it still can trigger automatic defensive processes (e.g., action preparation) mediated by the first network. Indeed, as one might expect in both the emotion- and color-naming task, participants responded slow to threatening compared with neutral actions (Pichon et al., 2012). We will come back to and extend this dynamic dual route perspective on affective perception in part II.

### Cognitive task load

The interaction between emotion and attention and the role of the AMG in this interaction is far from settled, as documented by the fMRI findings reported above. Moreover, the interpretation of these findings is complicated by several factors. First, fMRI measures emotion processing across a relatively long time-window. So, it is possible that initial encoding of emotions in the AMG is relatively independent from attention, but that top-down attention modulation is involved at later stages. A critical point for future research is therefore to “isolate” AMG activity in the earliest processing stages, which are more likely to occur in an automatic, pre-attentive, rather than controlled, resource-dependent fashion (Garrido et al., 2012). Also, task-related confounds may limit the interpretation of results. For example, in the Pessoa et al. (2002) study, participants judged the gender of the faces during the attended-faces trails, whereas they judged the orientation of peripheral bars during the unattended-faces trials. Thus, not only the focus of attention, but also the cognitive load, type of judgment and task varied across conditions, whereas in the study by Vuilleumier et al. (2001) these factors remained constant.

The studies we commented upon so far all compared two different tasks with different cognitive/attentional load to assess their influence on AMG activity. Yet this does not allow an assessment of task load *per se*. Indeed, the comparison is between the effect of two very different tasks, that of explicit conscious recognition and verbal naming of the emotion versus recognition and naming of another stimulus attribute unrelated to the emotion. This is a comparison between explicit recognition of emotion and explicit recognition of a non-emotion attribute. It is important to stress that we cannot rule out that in the so-called implicit condition participants may still be fully conscious of the stimuli and recognize the emotional valence while not reporting it simply because this is not part of the task. Under such conditions there may be AMG activity observed that is related not to the explicit stimulus and task demands but triggered by the stimuli independently of the task demands. Thus the term implicit does in fact cover a host of processes that are also possibly present in the explicit condition. For that reason it is imperative to unpack the notion of implicit in a number of different dimensions. One dimensions is task load, another is visual awareness. In this section and the next one, we discuss experiments where these different dimensions were addressed separately.

The goal of the next study (Sinke et al., 2012) was specifically to assess the importance of task difficulty itself and for that purpose we adapted the attention paradigm previously used (Sinke et al., 2010; Pichon et al., 2012) to allow both the manipulation of the focus of attention and attentional load. The former was manipulated by the use of new dynamic stimuli that depicted an angry conversation between two people, with an aggressor and a passive victim, and placing the colored dots on just one person. Attentional load was manipulated by using an easy or hard color-naming task. Thus the participants processed the same dynamic stimuli while paying attention to either the aggressor or passive victim under two attentional loads (low vs. high). Behaviorally there was no difference between the focus of attention factor during the hard color-naming, while in the easy task participants perform better when the focus of attention was on the aggressor. Consistent with previous results using dynamic stimuli and implicit tasks (Sinke et al., 2010; Pichon et al., 2012), deactivation of the AMG was observed. The left AMG showed an interaction and was less deactivated when the focus of attention was on the aggressor and not on the passive victim. This effect was strongest for the easy color-naming task (Sinke et al., 2012).

Recently, Shafer and colleagues provided for the first time evidence that supported both the view of Pessoa (2005) and Vuilleumier (2005). They used a perceptual discrimination task with emotional distracters and manipulated both the emotional charge of distracting information and the task demands. Results show that a wide variety of brain regions such as the dorsal medial and ventral lateral PFC are responsive to both task demands and emotional charge of the distracting stimuli. However, while AMG activation differentiated between high and low emotional distracting stimuli no difference was found in AMG activation under different task demands (Shafer et al., 2012). Another study found deactivation of the AMG when the static emotional face was not attended (Morawetz et al., 2010). Again, no difference was found between high and low attentional demands.

### Perception and visual unawareness

An important source of evidence concerning the role of the AMG in emotion processing comes from studies on patients with cortical blindness following destruction of the visual cortex. In fact, the lesion renders the patients clinically blind for the stimuli presented in the affected portion of the visual field (scotoma) and produces a pathological segregation between the major cortical route to the AMG, which is damaged, and the intact subcortical visual pathway, providing a unique experimental opportunity (Weiskrantz, 2009).

A recent behavioral/fMRI study in a patient (GY) with unilateral cortical blindness provides additional information on the effect of visual awareness on AMG activation (Van den Stock et al., 2011). While being clinically blind, GY performed above chance level in categorizing dynamic actions (same stimuli as used in Pichon et al., 2008). Furthermore, results show increased bilateral AMG activation besides superior colliculus (SC), pulvinar (Pulv), middle part of right fusiform gyrus (FG), and motor and somatosensory regions for non-consciously perceived angry actions compared with neutral actions. These results are consistent with both the literature on blindsight patients (Morris et al., 2001; Pegna et al., 2005; de Gelder and Hadjikhani, 2006) and non-conscious perception in healthy subjects (e.g., Whalen et al., 1998; Morris et al., 1999; Liddell et al., 2005).

Three findings are of relevance for the current review and the proposed dual route perspective of affective perception. First, cortical activation to non-conscious perception was restricted to the right FG, motor and somatosensory regions. Second, subcortical network activity was not found in the intact hemisphere associated with conscious perception of emotional actions. Third, cortical activation for conscious perception was observed in the prefrontal cortex (PFC), STS, precuneus and intraparietal sulcus (IPS). The results suggest that two separate neural systems underlie conscious and non-conscious perception. On the one hand, a geniculo-striate system underlies conscious perception and is mostly cortical based, while on the other hand, non-conscious perception seems based on the extrageniculo-striate and subcortical pathway including the AMG. However, several questions remain. Do these neural systems interact during the processing of emotional stimuli and what is the role of the AMG in both pathways?

## The role of the amygdala in a dual route perspective on affective perception

### The amygdala and attention: dual route, dual influence of attention?

In this final part we aim to recast some of the inconsistencies concerning the role of the AMG in relation to emotion and attention within the vantage point of a dynamic dual route perspective. Ultimately, a better understanding of the respective role of the different AMG subnuclei is needed.

Traditional face processing models view perception as starting with face categorization. Once this is successfully completed, it is followed by one or more successive stages of decoding the various face attributes like identity, emotion, gender, etc. In the framework of currently known brain areas that play a role in face processing this translates as initial categorization in occipitotemporal cortex (OFA) and STS, followed by fusiform face area (FFA), then extraction of the emotional valence following connections between FFA and AMG. Alternatively, we suggested that there may be separate processing routes already in the early stages, and this view is gaining momentum from new findings (e.g., de Gelder et al., 2001, 2003; de Gelder and Rouw, 2001; Meeren et al., 2005). Besides the ventral route, there is evidence for a dorsal route whereby affective information is rapidly extracted from incompletely processed stimuli. This non-ventral route may either depend on primary visual cortex (V1) processing, as argued in the classical picture of the dorsal route, or bypass that altogether and use subcortical structures such as the SC or the visual pulvinar as entry points.

In the area of body research a very similar picture dominates and perception is also viewed as following the ventral pathway. Researchers interested in neural representations of bodies and body parts have discovered two brain areas central to neurofunctional body representation, initially the extrastriate body area (EBA) and later the fusiform body area (FBA; Urgesi et al., 2004; de Gelder et al., 2010; Downing and Peelen, 2011). They have been attributed similar functions of category-specific visual processing well-known from face perception studies, encoding the details of the body stimulus or separate parts of body shape and fine detail of that body form (Downing and Peelen, 2011).

Variants of these roles are that EBA and OFA, respectively, code the stimulus part while only at the stage of FBA and FFA the whole stimulus is encoded (for a critique of this view see de Gelder et al., 2010). Urgesi et al. (2007) argued for a distinction between local versus configural processing of bodily stimuli and the involvement of the EBA in the former, whereas the superior parietal lobe and the ventral premotor area underlie the latter. However, both areas are nodes in a ventral processing route and both are assumed to come into play before so-called higher bodily attributes are processed. Indeed, similar to the view one finds in face perception theories, it is argued about EBA and FBA also that action, identity, and emotional state represent high-level information (de Gelder et al., 2010; Downing and Peelen, 2011). In contrast, when researchers address questions of affective perception and use bodies (or faces) representing an emotion, other structures that the ones belonging to the traditional ventral object recognition route emerge. In our approach, these structures can be grouped along a dorsal processing route.

**Figure 1:**
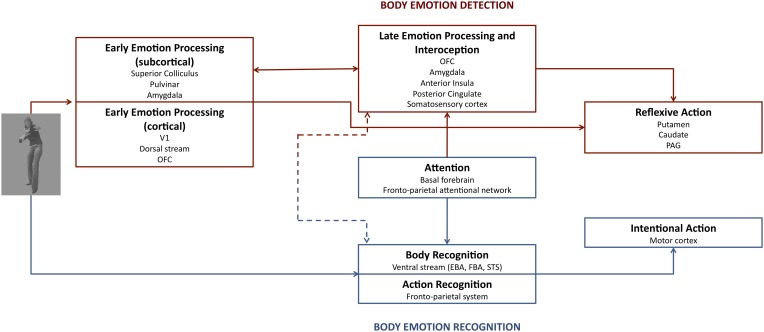
**The dual route of affective perception.** Emotion bodies are processed in two parallel routes with separate functions. Route I (red) underlies body emotion detection, whereas route II (blue) is important for body emotion detection. The amygdala plays a role in both early and late emotion processing. Attention has only an influence on late emotion processing and thus on amygdala activation. See text for further details.

Indeed, Vuilleumier (2005) already suggested a possibility of two pathways versus two stages for emotional control of perception. In this model, the AMG either receives directly or indirectly input from the SC and Pulv and provides feedback projection to the visual areas that projects to the AMG and cortical area (two-pathways hypothesis). Alternatively, the AMG receives coarse magnocellular inputs via a first feedforward sweep not mediated by the SC and Pulv, which is followed by reentrant feedback from the AMG, and further processing in the AMG and cortical areas (two-stage hypothesis). In our dynamic dual route of affective body perception, two routes (dorsal and ventral) underlie separate processes (see Figure 1). Route I consists of a subcortical (SC-Pulv-AMG) and cortical pathway (SC-Pulv-AMG-OFC) that sustains rapid automatic integration of affective content in the interest of adaptive reflex-like behavior (PAG, putamen, and caudate). This route is important for body detection, early emotional processing and reflexive action (de Gelder, 2006; Pichon et al., 2012). This dorsal processing route may or may not involve V1. For the sake of clarity, we refer to these two possibilities as the cortical and the subcortical dorsal route. Following the early emotional processing the slower late emotional processing occurs along with interoceptive mechanisms (AMG, OFC, posterior cingulate, anterior insula, and somatosensory cortex). This route is well-described in literature (e.g., Dalgleish, 2004; Vuilleumier, 2005; de Gelder, 2006), and the relevant findings are consistently replicated (Rudrauf et al., 2008; Garrido et al., 2012). A recent study used diffusion tensor imaging (DTI) to investigate *in vivo* anatomical connections between AMG and subcortical visual structures in a patient with early unilateral destruction of the visual cortex and healthy controls (Tamietto, Pullens et al., 2012). This study provides some unique evidence on the subcortical part of route I. First, they showed that the SC was connected with the AMG through the Pulv in both the patient and controls. Next, unilateral destruction of the visual cortex let to qualitative and quantitative modifications along these pathways within the damaged hemisphere. Fiber tracts between the AMG-Pulv and the SC-Pulv-AMG pathway were strengthened following ipsilateral V1 lesion, connections with frontal areas were reduced and new connections were formed between subcortical visual structures in the damaged hemisphere and posterior cortical areas in the opposite hemisphere (Tamietto, Pullens et al., 2012). This study suggests that two partially distinct anatomical and functional pathways mediate non-conscious and conscious emotion processing.

Route II lies parallel to route I and plays a role in body recognition (EBA, FBA, STS), action recognition (e.g., the fronto-parietal system), and attention. It is suggested that attention by means of activity of the fronto-parietal attentional network and the basal forebrain has a bidirectional relation with this route. The end result of route II is voluntary action (fronto-motor regions), although a shortcut exists in route I to trigger more reflexive action.

The findings that feed this debate on the relationship between AMG activity and attention may be best addressed in the context of a dynamic dual route model. In fact, while AMG is part of both routes, only route II appears modulated by attention and task constraints and has a direct impact on AMG activation.

### Dual route: fast processing of emotions and timing issues

Current methods in human affective neuroscience appear particularly limited to provide information about time course. Speed of processing is an important aspect of dual route claims. Initial evidence is coming from studies using the high temporal resolution of MEG. For example, activity in the Pulv (10–20 ms) and Amg (20–30 ms) is found for conscious perception of fearful expressions using MEG (Luo et al., 2007). As described above, this pattern of activation is frequently observed during non-conscious emotion perception with standard fMRI methods (Tamietto and de Gelder, 2010; Van den Stock et al., 2011). Besides early activity in the Pulv and AMG, cortical activity was observed in the visual cortex (40–50 ms) and in prefrontal areas (160–210 ms) (Luo et al., 2007). Another recent MEG study tested the dual route model to AMG, which predicts two parallel subcortical and cortical routes to AMG, against a model that excluded the subcortical pathway (Garrido et al., 2012). Results showed that the dual route model better explained activity to emotionally salient stimuli, and that this subcortical route was particularly important during early stages of stimulus processing.

However, in many fMRI studies subcortical activity during conscious emotion perception is often not observed. One explanation is that cortical feedback during conscious emotion perception might reflect inhibitory modulation over the subcortical SC-Pulv-Amg pathway (Tamietto and de Gelder, 2010). This is in line with the observation that non-conscious emotional perception can co-exist and interfere with conscious perception (Tamietto and de Gelder, 2008). Furthermore, when non-conscious perception is directly contrasted with conscious perception of emotions, relatively more activity in the Pulv and SC is observed in healthy subjects (Anderson et al., 2003; Bishop et al., 2004).

But independently of these methodological limitations and initial findings it is important to avoid theoretical misconception about the timing issue. It is in evolutionary terms more important which neural pathway supports quicker behavioral output (i.e., access to visuomotor integration and action) and not which brain area starts firing first in response to visual stimulation. Thus assuming a direct linear relationship between the latency of a neural response and the latency of a behavioral response is misleading. For example, speed of spontaneous expressive actions is faster for non-conscious emotion perception (Tamietto et al., 2009).

### Toward a better understanding of the parts to see the whole

In this review we considered the AMG as one homogenous structure, but this is certainly not the case. While an extensive discussion of the AMG subnuclei and the possible role of these structures are beyond the scope of this review, we will highlight several relevant aspects of the AMG anatomy and discuss these in terms of future research.

A widely accepted division of the human AMG is in terms of 3 main subnuclei, namely the basolateral (BLA), central-medial amygdala (CMA), and superficial amygdala (SFA; Heimer et al., 1997; Aggleton, 2000). These subnuclei have extensive afferent and efferent connections with almost all parts of the human brain and are strongly inter-connected by means of excitatory and inhibitory connections. Evidence from animal research suggests that each AMG subnuclei can be described in different terms (Davis and Whalen, 2001). The BLA and SFA complex are considered as the sensory input with the SFA specific for olfactory stimuli (Heimer et al., 1997), while the CMA is the behavioral output (for example see Mosher et al., 2010). As discussed above we state that the AMG plays an important role in both early and late processing of affective information. This rises the question what the roles of both the CMA and BLA are in these different aspects and the time course of emotional integration and, in addition, if AMG (de)activation is specific to certain nuclei. One can hypothesize, for example, that CMA, given the connection with hypothalamic and brain stem regions (Heimer et al., 1997), would be mostly active in early reflexive affective processing and less affected by attention. The BLA is more likely to be influenced by attention, given its importance in the integration of different visual cues and the afferent connections with the thalamus, other sensory regions and orbitofrontal cortex and efferent connections to the visual stream (Davis and Whalen, 2001). A somewhat related explanation of the intriguing but contrasting finding would be that early CMA activity is down-regulated by means of inhibitory connections with the BLA due to competing sensory information and thus that the implicit processing of affective stimuli is attenuated at a later stage. Unfortunately, few neuroimaging studies have used functional or structural localization of the different AMG subnuclei and thus the question if activity in AMG subnuclei is influenced by task demands or if the two subnuclei play different roles in the dual route remains unanswered. One important indication comes from the study by Terburg et al. (2012) who had the unique opportunity to test the role of AMG subnuclei in emotion processing in subjects with Urbach-Wiethe disease (UWD), a rare genetic disorder leading to bilateral calcification of the AMG. Using behavioral evidence, eye tracking, and structural and functional MRI measurements they propose that focal bilateral BLA damage with other AMG subnuclei remaining intact leads to hyper-vigilance for both non-conscious and dynamic fearful facial expressions. This provides some clues for the current debate on AMG activity and attention and the dual route of affective perception. It suggests that the CMA is most important for reflexive action as signified by hyper-vigilance to threat cues and thus is the critical node in early emotion processing via route I, whereas BLA could underlie, as suggested by Terburg et al. (2012), down-regulation of the threat vigilance system and reflexive action by means of inhibitory projections to the CMA and could be influenced by attention. Further research should investigate whether this is indeed the case.

## Conclusions

We have reviewed studies using dynamic bodily expressions and a variety of experimental setups to investigate the role of the AMG in emotion processing and the influence of attention on AMG activation. Taken together, we argue that in the dual route model of affective perception AMG activation can be observed in separate networks and at different time points. Both early and late emotion processing is partly supported by the AMG; however, only late AMG activation is influenced by attention.

With sophisticated paradigms and a wide variety of different stimuli (static or dynamic, emotional or non-emotional, facial or bodily expressions or social interactions) and the ever-expanding neuroscience toolbox one can only hope that after decades of research the question what AMG activity indicates will finally be answered.

### Conflict of interest statement

The authors declare that the research was conducted in the absence of any commercial or financial relationships that could be construed as a potential conflict of interest.
